# *CDH1* polymorphisms and haplotypes in sporadic diffuse and intestinal gastric cancer: a case–control study based on direct sequencing analysis

**DOI:** 10.1186/1477-7819-12-80

**Published:** 2014-03-31

**Authors:** Chi-Ming Chu, Cheng-Jueng Chen, De-Chuan Chan, Hurng-Sheng Wu, Yao-Chi Liu, Chen-Yang Shen, Tzu-Ming Chang, Jyh-cherng Yu, Horng-Jyh Harn, Cheng-Ping Yu, Ming-Hsin Yang

**Affiliations:** 1 Division of Biostatistics and Informatics, Department of Epidemiology, School of Public Health, National Defense Medical Center, No. 161, Sec. 6, Min-Quan E. Road, Neihu, Taipei 11490, Taiwan; 2 Division of General Surgery, Department of Surgery, Tri-Service General Hospital, National Defense Medical Center, No. 325, Sec. 2, Cheng-Kung Road, Neihu, Taipei 11490, Taiwan; 3 Division of General Surgery, Department of Surgery, Show Chwan Memorial Hospital, No. 6-1, Lugong Road, Lukang Township, Changhua 50005, Taiwan; 4 Institute of Biomedical Sciences, Academia Sinica, No. 128, Sec. 2, Academy Road, Nangang, Taipei 11529, Taiwan; 5 Division of General Surgery, Department of Surgery, Tungs’ Taichung MetroHarbor Hospital, No. 699, Sec. 8, Taiwan Blvd., Wuqi District, Taichung City 43502, Taiwan; 6 Department of Pathology, China Medical University Hospital, No. 2, Yuhder Road, Taichung 40447, Taiwan; 7 Department of Pathology, Tri-Service General Hospital, National Defense Medical Center, No. 325, Sec. 2, Cheng-Kung Road, Neihu, Taipei 11490, Taiwan

**Keywords:** *CDH1*, Direct sequencing, Haplotypes, Single-nucleotide polymorphisms, Sporadic diffuse and intestinal gastric cancer

## Abstract

**Background:**

Findings related to the influence of the −160C → A promoter polymorphism and haplotypes of the *E-cadherin* (*CDH1*) gene have not been consistent in previous studies regarding the risk for sporadic gastric cancer. Investigators in most previous studies detected those genotypes using restriction fragment length polymorphism analysis. Therefore, we conducted a case–control study to investigate the association of the *CDH1* − 160C → A promoter polymorphism and haplotypes for cancer risk related to sporadic diffuse and intestinal gastric cancer by direct sequencing analysis.

**Methods:**

We included 107 diffuse gastric cancer cases, 60 intestinal gastric cancer cases and 134 controls. The genotypic polymorphisms in the −160 promoter region, exons and intron–exon boundaries of *CDH1* were detected by direct sequencing analysis. Genotype frequencies were compared. The *CDH1* − 160C → A promoter polymorphism and four polymorphisms (48 + 6 T → C, 2076C → T, 2253C → T and 1937–13 T → C) were included in the haplotype analyses, which were estimated using the expectation–maximization algorithm.

**Results:**

Compared to controls, the frequency of the −160A allele was significantly higher in diffuse gastric cancer cases (*P* = 0.005), but it was not significantly different in intestinal gastric cancer cases (*P* = 0.119). Two sets of three-marker haplotypes (−160C → A, 48 + 6 T → C, 2076C → T and −160C → A, 1937–13 T → C, 2253C → T) were associated with the risk of diffuse gastric cancer (*P* = 0.011 and *P* = 0.042, respectively).

**Conclusion:**

Based on direct sequencing analysis, our findings suggest that the *CDH1* − 160C → A promoter polymorphism and haplotypes play significant roles in cancer risk for sporadic diffuse gastric cancer, but not for intestinal gastric cancer, in a Taiwanese population.

## Background

E-cadherin (encoded by the *CDHI* gene [dbSNP:NC_000016.9]) is a member of a family of transmembrane glycoproteins expressed in epithelial cells and is responsible for calcium-dependent cell-to-cell adhesion [1-3]. Loss of cell adhesion may contribute to loss of growth contact inhibition, which is an early step in the neoplastic process [4,5]. Furthermore, loss of cadherin activity may result in cancer cell detachment and metastasis [6]. Hence *CDHI* is considered to be a tumor suppressor gene.

A C/A single-nucleotide polymorphism (SNP) at the −160 position of the *CDH1* promoter region has been reported to result in downregulation of the transcription of this gene in a prostate cancer cell line, DU145, such that the A allele at this site decreased transcriptional efficiency by 68% compared with the C allele [7]. Therefore, the −160A allele variant has been considered to be a potential genetic marker for susceptibility to cancer. However, findings related to the influence of the −160C → A promoter polymorphism and haplotypes of the *E-cadherin* (*CDH1*) gene have not been consistent in previous studies regarding the risk for sporadic gastric cancer [8-16]. Some studies have reported that the *CDH1* − 160C → A promoter polymorphism was not found to be associated with the risk of gastric cancer [8-11], and others have reported that the −160A allele increased the risk for diffuse gastric cancer [12,13]. Conversely, two studies in Asian populations reported that the −160A allele decreased the risk of gastric cancer [15,16].

The genotypes of the −160C → A promoter polymorphism in most of the previous studies were detected by restriction fragment length polymorphism (RFLP) analysis [8-13,15,16]. RFLP analysis is an indirect method applied extensively in the past [17]. However, it involves a manual process that is based on gel-based processing techniques and the results it produces are subjectively evaluated by direct observation. In contrast, direct sequencing is a standardized procedure, and the results are processed by a computer [18].

By literature review, we compared the *CDH1* − 160A allele frequency in controls in studies in which RFLP analysis was used, and the −160A allele frequency in healthy samples reported by HapMap based on direct sequencing derived from the National Center for Biotechnology Information dbSNP Short Genetic Variations online database [19]. The frequency of the −160A allele was calculated using the formula CA% × ½ + AA%, where CA is the C and A alleles and AA is the double A alleles. Humar *et al.* reported that the frequency of the −160A allele in controls in an Italian population was 23.6% [12], and Corso *et al*. reported a frequency of 32.05% in a different Italian population [11]. In a study by Pharoah *et al*., the frequency of the −160A allele was found to be 30% for Canadian, German and Portuguese populations [8]. Park *et al*. reported that the frequency of the −160A allele in controls in a Korean population was 23% [9]. Lu *et al*. reported that the frequency of the −160A allele was 24.3% for Chinese individuals in Jiangsu Province, China [10]. Medina-Franco *et al*. reported that the frequency of the −160A allele was 24.35% in a Mexican population [13]. Wu et al. reported that the frequency of the -160A allele was 33.67% in a Taiwanese population [15]. Kuraoka *et al*. reported that the frequency of the −160A allele was 35% in a Japanese population [16]. The HapMap database, however, gives information based on direct sequencing that the frequency of the −160A allele in healthy samples is 26.0% for Europeans in Italy, 25.2% for Han Chinese in Beijing, 19.9% for Japanese in Tokyo and 15.0% for Africans in Nigeria [19]. In aforementioned studies in which RFLP analysis was used, the reported frequencies of the −160A allele in controls were diverse within the same ethnic groups, and some were much higher than the values in the HapMap database. Therefore, genotype detection by RFLP analysis in some of previous studies might be inaccurate, which might contribute to the inconsistency of results in previous studies. Tanahashi *et al*. and Davis *et al*. also reported that RFLP analysis was less accurate than direct sequencing in their studies [20,21]. However, researchers in many studies have suggested that environmental factors, lifestyles and ethnic differences might account for opposite directions in associations of the *CDH1* − 160C → A polymorphism with gastric cancer among some Asian and Caucasian studies [8-14,22,23]. Gene-based haplotypes, which are collections of SNPs located throughout the functional regions of candidate genes, may have greater power than any individual polymorphism in influencing a clinical response [24].

In one study of haplotype analysis using RFLP methods, the investigators suggested that the *CDH1* − 160C → A polymorphism might be in linkage disequilibrium with other distinct *CDH1* polymorphisms in sporadic diffuse gastric cancer [12] Researchers in another study using direct sequencing reported a statistically nonsignificant risk of −160C → A polymorphism containing haplotypes associated with gastric cancer without differentiation of histopathologic subgroups [14]. Furthermore, diffuse and intestinal types of gastric cancer are different in terms of their epidemiology, etiology, pathogenesis and behavior [25]. Therefore, we investigated the influence of the *CDH1* − 160C → A promoter polymorphism and haplotypes on risk for diffuse and intestinal gastric cancer separately by direct sequencing.

## Methods

### Patients and samples

Eligible cases comprised 167 sporadic gastric cancer patients who had undergone surgical treatment between 2001 and 2005 in the Division of General Surgery, Department of Surgery, Tri-Service General Hospital, Taipei, Taiwan. All of the Taiwanese patients were Han Chinese, but no aborigines were included, as they belong to a different ethnic group that account for only 0.02% of the population in Taiwan. None of the patients had familial gastric cancer. All patient DNA obtained from the noncancerous gastric epithelia of resected specimens were stored in liquid nitrogen. Control DNA was obtained from the peripheral blood of 134 healthy individuals who attended general health checkup including upper gastrointestinal tract endoscopy in this hospital. The study participants provided written informed consent prior to participation. The study was approved by the Institutional Review Board of the Tri-Service General Hospital. All surgical samples were classified into diffuse and intestinal types according to the Laurén criteria [25].

### Immunohistochemical staining and evaluation of E-cadherin expression

Specimens from paraffin blocks were cut into 5-μm sections and stained with hematoxylin and eosin for histological diagnosis. E-cadherin expression was evaluated using a monoclonal antibody (Cappel, Aurora, OH, USA) followed by a secondary antibody [26]. The signal was detected using an avidin-biotin complex and a 3,3′-diaminobenzidine (DAB) kit (Vector Laboratories, Burlingame, CA, USA). DAB produced a yellowish brown stain if a sample was positive. If more than 90% of the tumor cells showed intense membranous staining similar to that of normal cells, the result was considered positive (++). If the staining intensity was demonstrably reduced relative to that of normal cells and/or if the staining pattern was heterogeneous (10% to 90% positive), the result was recorded as weakly positive (+). If immunohistochemical expression was completely absent or positive in less than 10% of the cells, the result was defined as negative (−).

### Genotyping

DNA samples from all cases were extracted from noncancerous gastric epithelia. Control DNA samples were isolated from peripheral blood lymphocytes by proteinase K digestion and phenol-chloroform extraction. All CDH1 exons and the corresponding intron–exon boundaries of case DNA and control DNA were amplified using primers developed by Berx *et al*. [27]. The primers used to amplify the −160 promoter region were described by Li *et al*. [7]. PCR was carried out in volumes of 25 μl containing 20 ng of genomic DNA as a template, 2 mM MgSO4, 0.4 μM sense and antisense primers, 0.25 mM deoxyribonucleotide triphosphate, and buffer containing *Taq* DNA polymerase. The amplification program was as follows: 40 cycles with a denaturing temperature of 95°C for 30 seconds, annealing for 30 seconds and extension at 72°C for 30 seconds in a DNA thermal cycler. All amplified products were purified, and direct sequencing was performed using an ABI PRISM 377 automated sequencer and an ABI PRISM Dye Terminator Cycle Sequencing Kit (PerkinElmer, Greenville, SC, USA).

### Statistical analysis

Stata 8 software (StataCorp, College Station, TX, USA) was used to manage data and for statistical analyses. The observed genotype frequencies were compared between cases and controls using a χ^2^ test. Fisher’s exact test was used to assess the genotype and allele distributions in diffuse and intestinal type cases. The allelic distribution was in Hardy-Weinberg equilibrium in the case and control groups (*P* > 0.82). Genotype-specific risks were estimated as the odds ratio (OR) with associated 95% confidence interval (CI) by unconditional logistic regression. ORs were adjusted according to age and gender. A *P*-value less than 0.05 was considered statistically significant. The study power was 0.7758 to 0.9509 with an α of 0.05 [28]. The haplotype frequencies for various marker combinations were estimated separately for the two type cases and the controls by using an expectation-maximization algorithm.

## Results

### Characteristics of diffuse and intestinal type cases

**Figure 1:**
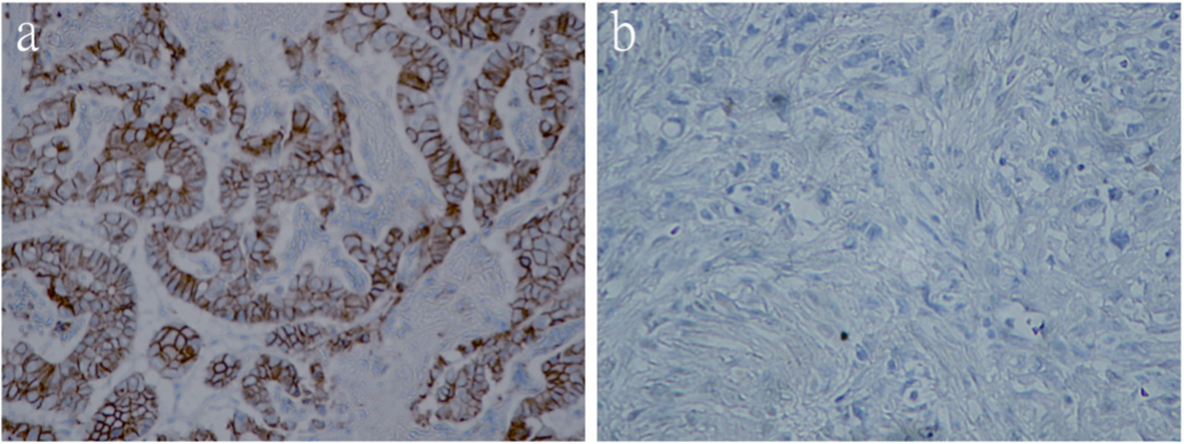
**Immunohistochemical staining for E-cadherin. (a)** E-cadherin-positive (++) in intestinal type cancer. **(b)** E-cadherin-negative (−) in diffuse type cancer.

The 134 controls were composed of 86 males and 48 females. Their mean age was 51.06 ± 13.04 years (range = 19 to 89 years). The 167 gastric cancer cases comprised 114 males and 53 females. Their mean age was 69.11 ± 13.04 years (range = 27 to 90 years). According to Laurén’s classification system, 107 cases were of the diffuse type and 60 cases were of the intestinal type. The mean age of the diffuse type cases was 66.87 ± 13.81 years (range = 27 to 90 years) and that of the intestinal type cases was 73.82 ± 9.76 years (range = 48 to 88 years). The mean age of the diffuse type cases was approximately 7 years younger than that of the intestinal type cases. No differences were observed with respect to gender or TNM stages I to III in these two types. There were significantly more diffuse gastric cancer cases classified as stage IV. Reduced E-cadherin expression (Figure 1) was more frequent in the diffuse type cases than in the intestinal type cases. Comparison of the characteristics between these two case types is summarized in Table 1.

**Table 1:** Comparison of clinicopathologic characteristics between intestinal and diffuse type tumors

**Characteristics**	**Intestinal type, *n *(%) (*N*** **= 60)**	**Diffuse type, *n *(%) (*N*** **= 107)**	***P-*** **value**
Gender			
Male	46 (76.7%)	68 (63.6%)	0.081^a^
Female	14 (23.3%)	39 (36.5%)	
Age (yr)			
Mean ± SD	73.82 ± 9.76	66.87 ± 13.81	0.0001^b^
TNM staging			
Stage I	16 (26.7%)	16 (15.0%)	0.065^c^
Stage II	8 (13.3%)	24 (22.4%)	0.152^c^
Stage III	28 (46.7%)	35 (32.7%)	0.074^c^
Stage IV	8 (13.3%)	32 (30.0%)	0.016^c^
Reduced E-cadherin expression	32 (53.3%)	93 (86.9%)	<0.001^c^

^a^χ^2^ test. ^b^*t*-test. ^c^Proportion test.

### Genotype detected by direct sequencing

**Figure 2:**
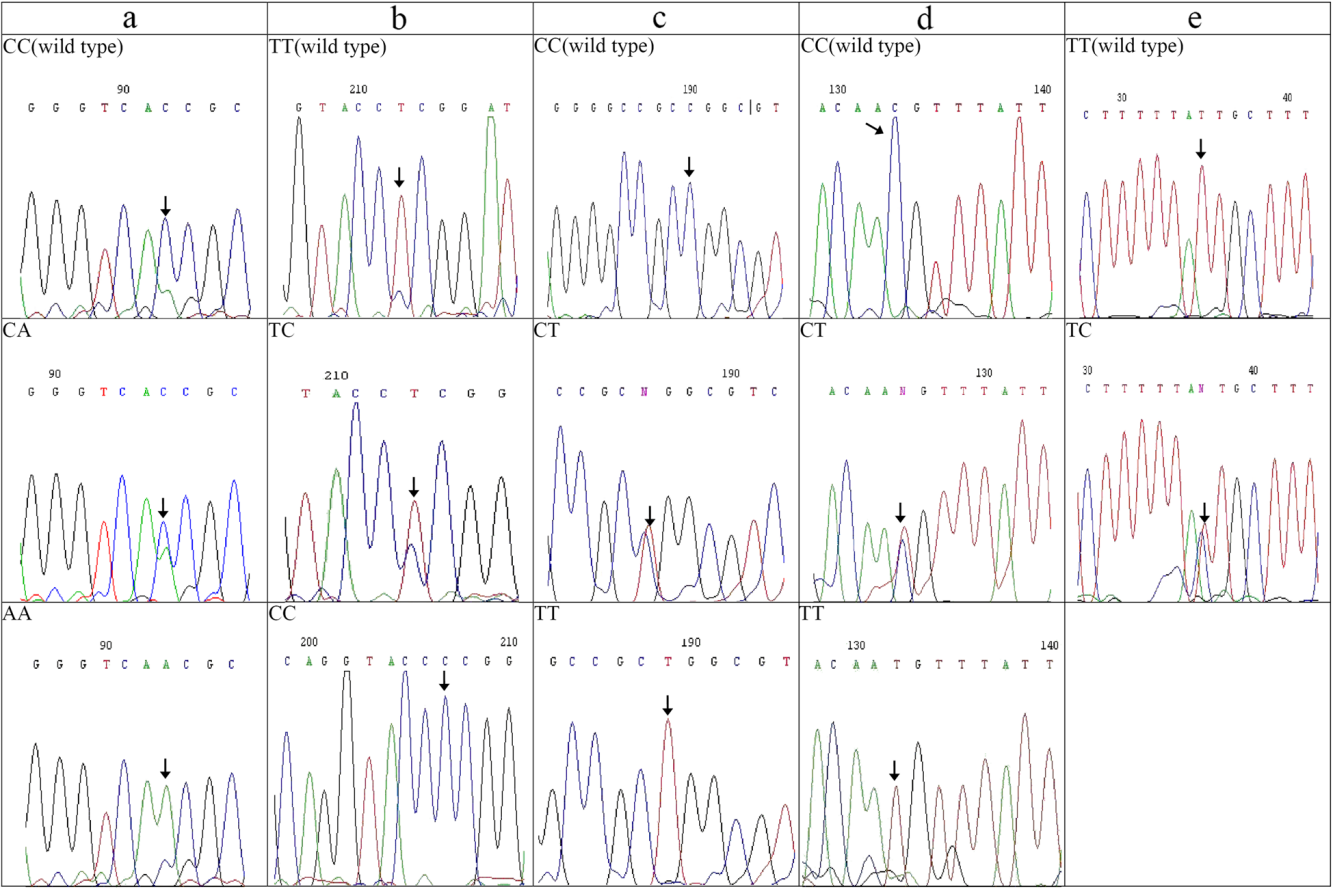
**Genotypes of *CDH1 *polymorphisms detected by direct sequencing. (a)** Promoter polymorphism −160C → A. **(b)** Intron 1 polymorphism 48 + 6 T → C. **(c)** Exon 13 polymorphism 2076C → T. **(d)** Exon 14 polymorphism 2253C → T. **(e)** Intron 12 polymorphism 1937–13 T → C.

The genotypes of the *CDH1* − 160C → A (dbSNP:rs16260), 48 + 6 T *→* C (dbSNP:rs3743674), 2076C → T (dbSNP:rs1801552), 2253C → T (dbSNP:rs33964119) and 1937–13 T → C (dbSNP:rs2276330) polymorphisms detected by direct sequencing in the cases and controls are shown in Figure 2.

### Single-locus analysis in diffuse type cases

For promoter polymorphism −160C → A, the frequency of the −160A allele was significantly higher in diffuse type cases compared to the controls (*P* = 0.005). The OR associated with the A allele was 1.750 (95% CI = 1.014 to 3.022) for CA heterozygotes and 4.375 (95% CI = 1.467 to 14.565) for AA homozygotes. For other four polymorphisms (48 + 6 T → C, 2076C → T, 1937–13 T → C and 2253C → T), the genotype frequencies were not significantly different between the diffuse type cases and the controls (*P* = 0.441, 0.649, 0.147 and 0.982, respectively) (Table 2).

**Table 2:** **Genotype frequencies of *CDH1 *** **polymorphisms in diffuse gastric cancer cases and controls**

**Variant**	**Cases vs. controls**	***n***	**(%)**	***n***	**(%)**	***n***	**(%)**	***n***	**(%)**	**Statistical tests**		**Fisher’s exact test**
										**χ**^**2 **^**test**	***P*-value**	
Promoter		CC		CA		AA		Total		10.7858	0.005	0.005
−160C → A	Diffuse	48	44.86%	44	41.12%	15	15.00%	107	100.00%			
	Controls	84	62.69%	44	32.84%	6	4.48%	134	100.00%			
Odds ratio^a^		1		1.750 (1.014 to 3.022)		4.375 (1.467 to 14.565)						
Intron 1		TT		TC		CC				1.6397	0.441	0.489
48 + 6 T → C	Diffuse	71	66.36%	32	29.91%	4	3.74%	107	100.00%			
	Controls	92	68.66%	33	24.63%	9	6.72%	134	100.00%			
Odds Ratio^a^		1		1.257 (0.677 to 2.328)		0.576 (0.125 to 2.170)						
Exon 13		CC		CT		TT				0.8651	0.649	0.641
2076C → T	Diffuse	45	42.06%	48	44.83%	14	13.08%	107	100.00%			
	Controls	55	41.05%	66	49.25%	13	9.70%	134	100.00%			
Odds ratio^a^		1		0.889 (0.499 to 1.584)		1.136 (0.514 to 3.381)						
Intron 12		TT		TC		CC				2.100	0.147	0.182
1937-13 T → C	Diffuse	89	83.18%	18	16.82%	0	0.00%	107	100.00%			
	Controls	120	89.55%	14	10.45%	0	0.00%	134	100.00%			
Odds ratio^a^		1		1.734 (0.766 to 3.976)		to						
Exon 14		CC		CT		TT				0.037	0.982	1.000
2253C → T	Diffuse	89	83.18%	17	15.89%	1	0.93%	107	100.00%			
	Controls	111	82.84%	22	16.42%	1	0.75%	134	100.00%			
Odds ratio^a^		1		0.964 (0.451 to 1.842)		1.247 (0.016 to 98.766)						

^a^Adjusted for age and sex.

### Single-locus analysis in intestinal type cases

In the intestinal type cases, no significant association with disease was found for any of the five polymorphisms (−160C → A, 48 + 6 T → C, 2076C → T, 1937–13 T → C and 2253C → T; *P* = 0.119, 0.329, 0.185, 0.889 and 0.375, respectively).

### Haplotype analysis in diffuse type cases

A three-marker haplotype (−160C → A, 48 + 6 T → C, 2076C → T) showed a significant association with disease and the ATC, ACC haplotypes were found to be associated with increased risk for diffuse type cancer (Table 3). A different three-marker haplotype (−160C → A, 1937–13 T → C, 2253C → T) also exhibited a significant association with disease and the ATC haplotypes was associated with increased risk for diffuse gastric cancer. The two other sets of three-marker haplotypes (−160C → A, 2076C → T, 2253C → T and −160C → A, 48 + 6 T → C, 1937–13 T → C) exhibited no significant association with disease (Table 3).

**Table 3:** ***CDH1 *** **three-marker haplotype analysis in diffuse gastric cancer cases**

**Three polymorphisms**	**Haplotype**	**Cases (%)**	**Controls (%)**	**OR**	**Lower 95% CI**	**Upper 95% CI**	***P*-value**
−160C → A	CTT	24.70	25	1.132	0.860	1.489	0.011
48 + 6 T → C	ATT	5.93	4.48	1.511	0.926	2.466	
2076C → T	CCC	12.07	13.27	1.030	0.731	1.452	
	CTC	31.78	36.38	1			
	CCT	3.80	4.48	0.954	0.552	1.648	
	ATC	18.81	15.11	1.431	1.051	1.949	
	ACC	2.52	0.93	3.272	1.322	8.096	
	ACT	0.38	0.37	0.954	0.158	5.761	
−160C → A	CTT	7.72	8.02	1.079	0.729	1.597	0.042
1937-13 T → C	ATT	1.41	0.75	2.073	0.732	5.872	
2253C → T	CCC	3.20	3.36	1.075	0.599	1.929	
	CTC	61.22	67.53	1			
	CCT	0.21	0.19	0.691	0.062	7.648	
	ATC	23.34	18.47	1.4	1.078	1.819	
	ACC	2.86	16.79	0.184	0.111	0.306	
	ACT	0.04	0	–	–	–	
−160C → A	CCT	1.62	14.93	0.108	0.055	0.212	0.096
2076C → T	ACT	0.09	0.19	0.691	0.058	7.227	
2253C → T	CTC	37.54	42.91	0.909	0.703	1.176	
	CCC	26.88	27.99	1			
	CTT	6.31	6.72	0.987	0.628	1.551	
	ACC	6.23	4.66	1.406	0.862	2.293	
	ATC	19.97	15.49	1.332	0.971	1.827	
	ATT	1.37	0.56	2.341	0.771	7.111	
−160C → A	CTC	53.84	58.79	0.904	0.470	1.739	0.103
48 + 6 T → C	ATC	22.01	17.91	1.21	0.613	2.389	
1937-13 T → C	CCT	0.77	0.93	0.882	0.237	3.281	
	CTT	2.65	2.61	1			
	CCC	15.10	16.79	0.888	0.445	1.772	
	ATT	2.73	1.68	1.588	0.616	4.094	
	ACT	0.17	0	–	–	–	
	ACC	2.73	1.31	2.021	0.754	5.416	

### Haplotype analysis in intestinal type cases

In the intestinal type cases, no significant association with disease was found for any of the four sets of three-marker haplotypes (−160C → A, 48 + 6 T → C, 2076C → T; −160C → A, 1937–13 T → C, 2253C → T; −160C → A, 2076C → T, 2253C → T and −160C → A, 48 + 6 T → C, 1937–13 T → C; *P* = 0.164, 0.319, 0.408 and 0.607, respectively).

## Discussion

To detect SNP variations, both direct sequencing and RFLP are suitable. RFLP is subject to experimental problems, however, such as incomplete digestion and relatively poor resolution, resulting in misclassification of genotypic status. Nevertheless, many previous studies have depended on RFLP because it is cheaper and easy to perform. However, the cost of direct sequencing has decreased significantly, and regular automatic sequencing platforms are able to provide reliable results easily. The use of sequencing in the present study can be considered a major strength.

In this study, specimens were obtained from 167 Taiwanese patients with sporadic gastric cancer who underwent surgical treatment. The patients with intestinal gastric cancer are generally older, have more comorbidities and tend not to undergo surgical treatment, which accounts for more diffuse gastric cancer cases than intestinal gastric cancer cases. Although we used an acceptable number of cases with statistical power, the sample size was still relatively small. Further studies including more cases are needed.

The frequency of the A allele in the *CDH1* − 160C → A promoter polymorphism in this study was significantly greater in diffuse type cases than in the controls. Humar *et al*. [12] and Medina-Franco *et al*. [13] reported similar results.

It has been suggested that *CDH1* may contribute to gastric cancer risk in a complex manner due to multiple polymorphic variants [12,14]. Humar *et al*. confirmed that the three-marker haplotype (−160C → A, 48 + 6 T → C, 2076C → T) was associated with diffuse gastric cancer and suggested that haplotype ATT (−160A, 48 + 6 T, 2076 T) was a marker for diffuse gastric cancer susceptibility, whereas haplotype CTT had a protective effect [12].

We also found that the three-marker haplotype (−160C → A, 48 + 6 T → C, 2076C → T) was associated with diffuse gastric cancer and the ATC and ACC haplotypes were associated with increased risk. However, the haplotype CTT had no protective effect on diffuse gastric cancer which differs from that reported by Humar *et al*. [12]. Furthermore, we found another three-marker haplotype (−160C → A, 1937–13 T → C, 2253C → T) that was associated with diffuse gastric cancer, and the ATC haplotypes showed increased risk.

In the three-marker haplotype in this study (−160C → A, 48 + 6 T → C, 2076C → T), the haplotype ATC, with only one polymorphic variant (−160A) showed increased diffuse gastric cancer risk. Therefore, with respect to the *CDH1* − 160C → A polymorphism, the A allele might result in a defect in gene transcription and increased risk for diffuse type cancer. The ACC haplotype, with two polymorphic variants (−160A and 48 + 6C), exhibited a higher OR. This finding indicates that the presence of two polymorphic variants in this haplotype produces synergic effects related to cancer risk.

## Conclusions

We conclude that, based on direct sequencing analysis, the −160C → A promoter polymorphism and the investigated haplotypes play significant roles in the risk for sporadic diffuse gastric cancer, but not for intestinal gastric cancer, in a Taiwanese population. In the future, additional polymorphisms in other regions of the same gene, such as the promoter region and splicing sites, should be evaluated, and haplotype analysis should include additional polymorphisms for the purpose of increasing knowledge about how combinations of polymorphisms can influence cancer risk.

## Abbreviations

RFLP: Restriction fragment length polymorphism; SNP: Single-nucleotide polymorphism.

## Competing interests

The authors declare that they have no competing interests.

## Authors’ contributions

LYC and SCY designed the research. LYC, CCJ, CDC, CCM, WHS, CTM, YJC, HHJ, YCP and YMH performed the research and analyzed the data. CCM performed the statistical analysis. LYC, CCJ and CDC wrote the paper. All authors read and approved the final manuscript.
